# Liquid–liquid phase separation of amyloid-β oligomers modulates amyloid fibrils formation

**DOI:** 10.1016/j.jbc.2023.102926

**Published:** 2023-01-20

**Authors:** Xinrui Gui, Shuang Feng, Zilong Li, Yanyan Li, Bernd Reif, Bingyang Shi, Zheng Niu

**Affiliations:** 1 Henan-Macquarie University Joint Centre for Biomedical Innovation, School of Life Sciences, Henan University, Kaifeng, China; 2 Henan Key Laboratory of Brain Targeted Bio-nanomedicine, School of Pharmacy, Henan University, Kaifeng, China; 3 Munich Center for Integrated Protein Science (CIPS-M) at the Department of Chemistry, Technische Universität München (TUM), Garching, Germany; 4 Deutsches Forschungszentrum für Gesundheit und Umwelt Institute of Structural Biology, Helmholtz-Zentrum München (HMGU), Neuherberg, Germany; 5 Macquarie Medical School, Faculty of Medicine & Health Sciences, Macquarie University, Sydney, NSW, Australia

**Keywords:** amyloid-β oligomers, liquid–liquid phase separation, oligomerization, protein aggregation, Alzheimer’s disease, AβO, amyloid-β oligomers, AD, Alzheimer’s disease, DMSO, dimethyl sulfoxide, EGCG, epigallocatechin gallate, FRAP, fluorescence recovery after photobleaching, HFIP, hexafluoroisopropanol, HiPPS, high-throughput protein phase separation, LLPS, liquid–liquid phase separation, MW, molecular weight, ROI, region of interests, TBST, Tris buffered saline with Tween-20, TDP-43, TAR DNA-binding protein of 43 kDa, TEM, transmission electron microscopy, ThT, thioflavin-T

## Abstract

Soluble amyloid-β oligomers (AβOs) are proposed to instigate and mediate the pathology of Alzheimer’s disease, but the mechanisms involved are not clear. In this study, we reported that AβOs can undergo liquid–liquid phase separation (LLPS) to form liquid-like droplets *in vitro*. We determined that AβOs exhibited an α-helix conformation in a membrane-mimicking environment of SDS. Importantly, SDS is capable of reconfiguring the assembly of different AβOs to induce their LLPS. Moreover, we found that the droplet formation of AβOs was promoted by strong hydrated anions and weak hydrated cations, suggesting that hydrophobic interactions play a key role in mediating phase separation of AβOs. Finally, we observed that LLPS of AβOs can further promote Aβ to form amyloid fibrils, which can be modulated by (−)-epigallocatechin gallate. Our study highlights amyloid oligomers as an important entity involved in protein liquid-to-solid phase transition and reveals the regulatory role of LLPS underlying amyloid protein aggregation, which may be relevant to the pathological process of Alzheimer’s disease.

The aberrant aggregation of proteins from soluble state to insoluble amyloid fibrils is linked to the pathogenesis of neurodegenerative diseases, including Alzheimer’s disease (AD), amyotrophic lateral sclerosis, Parkinson’s disease, and type 2 diabetes (1, 2, 3). In addition to misfolding and aggregation, many of these disease-associated proteins undergo liquid–liquid phase separation (LLPS) to form dynamic condensates, which play a fundamental role in normal cellular processing (4, 5, 6, 7). For example, Alzheimer’s-associated tau protein forms liquid droplets at physiological protein levels *in vitro* and the droplet formation has an important physiological function to increase the local concentration of tubulin and further to nucleate the formation of microtubule bundles (8). Similarly, LLPS has also been observed during the α-synuclein aggregation and the liquid-to-solid phase transition yields amyloid hydrogels which are associated with cellular toxicity (6). Islet amyloid polypeptide undergoes LLPS process that is catalyzed by hydrophobic–hydrophilic interfaces (*e.g.*, air-water interface *in vitro*) and the LLPS-driven aggregation process is important to the pathology of type 2 diabetes (7). In addition, fused in sarcoma (FUS), heterogeneous nuclear ribonucleoprotein A1 (hnRNPA1), and TAR DNA-binding protein of 43 kDa (TDP-43) are found in the pathological inclusions of amyotrophic lateral sclerosis patients participating in stress granule formation *via* LLPS to regulate the metabolism of RNA (9, 10, 11).

LLPS is a reversible process of protein assembly into dynamic micro-sized liquid-like droplets. The intrinsically disordered regions, low complexity domains, and repetition motifs are prone to facilitate phase separation. LLPS is driven by multivalent weak interactions that primarily include electrostatic interactions (12, 13), π–π and cation–π interactions (14), and hydrophobic interactions (15) etc. Protein LLPS actively contributes to amyloid fibrils maturation and deposition, in which the droplets can further maturate into less-dynamic structures and finally yield irreversible pathological amyloid fibrils (11, 16, 17). In mature condensates formed *in vitro*, amyloid-like filaments can be observed and share similar morphology and structure with the pathological assemblies of neurodegenerative diseases (9, 11, 18, 19), indicating this aberrant liquid-to-solid phase transition may be the early event involved in neurodegeneration (20). Therefore, targeting LLPS to block phase transition and protein aggregation while maintaining cellular physiological functions will lead to potential therapeutics for neurodegenerative diseases.

Amyloid proteins initially self-assemble into various oligomers and associate to form amyloid fibrils in a nucleation pathway (3). This protein aggregation can be normally regarded as sequential oligomerization process in discrete. Assemblies formed by oligomerization are important intermediates which exert cytotoxicity relevant to human diseases (21). Oligomerization was reported to be related to protein LLPS. The toxic oligomers of amyloid proteins like α-synuclein and tau are found in the phase-separated state of proteins (5, 6), and the oligomerization of the N-terminal domain of TDP-43 has been demonstrated to enhance the phase separation of full-length TDP-43 (22). However, whether amyloid oligomers exhibit LLPS behavior and how it links to pathological amyloid aggregation are poorly understood. In this work, we observed the phase separation of amyloid-β oligomers (AβOs) through a high-throughput protein phase separation (HiPPS) profiling method (23). AβOs were prepared in SDS environment which reformed the assembly state of oligomers that enabled phase separation. Meanwhile, we found that AβOs LLPS modulated a liquid-to-solid phase transition and redirected Aβ aggregation pathway to form amyloid fibrils. Our finding provides further insights into the mechanism of how Aβ oligomerization regulates protein LLPS and liquid-to-solid phase transition.

## Results

### Aβ_42_Os undergo LLPS in vitro

**Figure 1:**
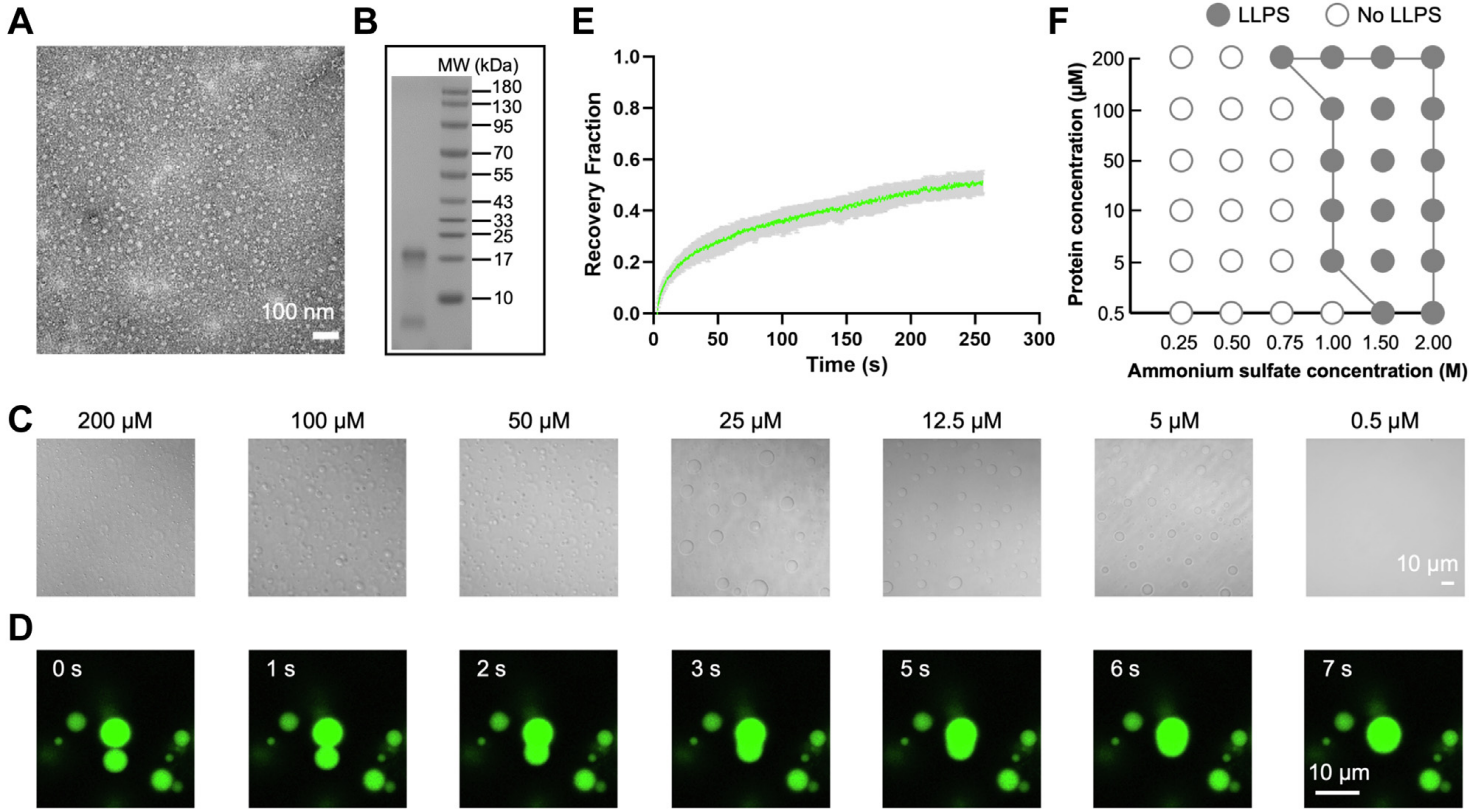
**Aβ**_**42**_**Os undergo LLPS *in vitro*.***A*, negative-staining TEM image of 200 μM Aβ_42_Os in 20 mM NaH_2_PO_4_, 140 mM NaCl buffer, pH 7.4 containing 0.2% (w/v) SDS. *B*, Aβ_42_ oligomerization studied by Coomassie staining Tricine-SDS-PAGE. *C*, liquid-like droplets of Aβ_42_Os observed under light microscope with various concentrations. *D*, representative images of droplet fusion over time. *E*, molecule rearrangement within droplets assessed by FRAP assay. Data were shown as means ± S.D. with n = 5 individual droplet. *F*, phase diagram of Aβ_42_Os under different concentrations of ammonium sulfate. *Solid circle* indicates phase separation, and *hollow circle* indicates no phase separation. FRAP, fluorescence recovery after photobleaching; LLPS, liquid–liquid phase separation; TEM, transmission electron microscopy.

Low molecular weight (MW) globular oligomers of Aβ_42_ (Aβ_42_Os) in 0.2% (w/v) SDS were prepared as previously described (24). During Aβ_42_Os preparation, dimethyl sulfoxide (DMSO) solvent is introduced to dissolve Aβ_42_ peptide film (for detailed information, see Experimental procedures). To exclude the interference of DMSO on protein stability and aggregation, and to further examine the effects of peptide film dissolution and buffer conditions on oligomer species, we dissolved Aβ_42_ peptide film either in DMSO solvent (Protocol I) or in a DMSO-free condition with NaOH solution (Protocol II). Protein electrophoresis, transmission electron microscopy (TEM) imaging technique, and CD measurement were performed to evaluate the oligomer properties including MW, morphology, and secondary structure. The results (Fig. S1, *A*–*D*) are demonstrated that these two different preparation protocols and buffer conditions yield similar Aβ_42_ oligomer species. Spherical oligomers were observed with a diameter of ∼10 nm from TEM (Fig. 1*A*). The band with MW of ∼18 kDa was displayed in Tricine-SDS-PAGE, in corresponding to a tetramer formation (Fig. 1*B*). After a large number of screenings with HiPPS profiling method (23), liquid droplets of Aβ_42_Os were observed in 50 mM Tris/HCl pH 8.5 aqueous buffer containing 1 M ammonium sulfate. LLPS of Aβ_42_Os occurred within a wide range of concentrations between 5 μM ∼ 200 μM, where Aβ_42_ formed stable oligomers (Figs. 1*C* and S1*E*). The droplet formation of Aβ_42_Os depends on protein and salt concentrations (Fig. 1*F*). Both synthetic and recombinant Aβ_42_ monomeric peptides were applied for further Aβ_42_Os preparation, and the results suggest that Aβ_42_Os prepared from different sources formed similar liquid droplets under the above condition (Fig. S1*F*).

To study the liquid properties of the droplet formed by Aβ_42_Os, N-terminal labeled FITC-Aβ_42_ peptide was synthesized to prepare fluorescent labeled oligomers for observation. FITC-Aβ_42_Os were mixed with unlabeled Aβ_42_Os at the molar ratio of 1:25. We found that two small droplets spontaneously fused into one large-size droplet within seconds under fluorescence microscope (Fig. 1*D*), which indicates liquid behavior of protein dense phase. Additionally, the mobility of the droplet was assessed by fluorescence recovery after photobleaching (FRAP) measurement. The fluorescence signal recovered up to 60% after bleaching a small area (∼2 μm circle in diameter) inside a single droplet (Fig. 1*E*), which suggests the rapid rearrangement of the molecules inside the protein dense phase. The relatively low FRAP recovery reflects the less-dynamic property of the liquid-like droplets formed by Aβ_42_Os, which may be due to the high viscosity nature of the droplet formed by Aβ_42_Os or the aggregation of amyloid proteins inside the droplets.

### SDS reshapes the assembly of Aβ_42_Os that enables LLPS

**Figure 2:**
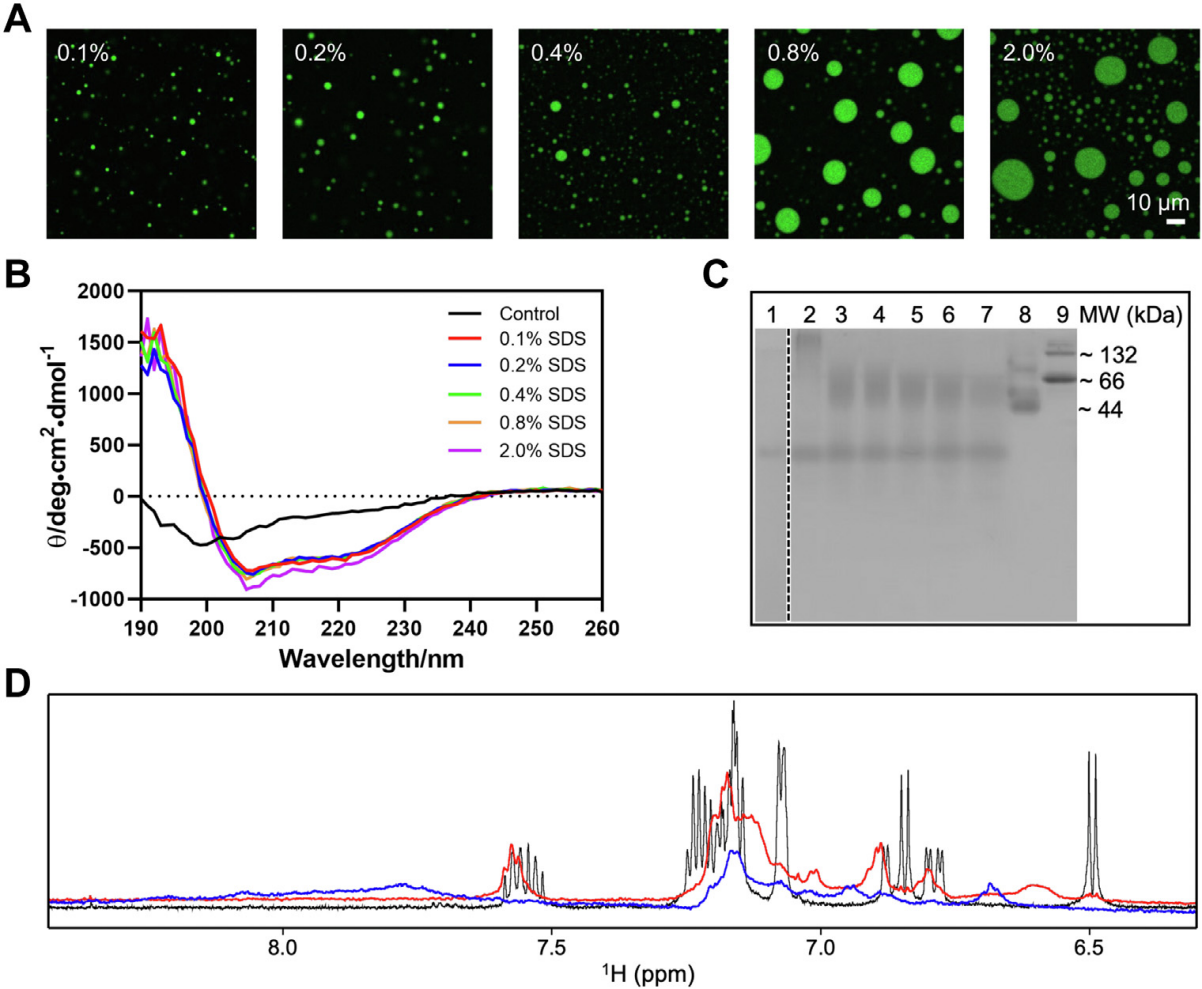
**SDS reshapes the assembly of Aβ**_**42**_**Os that enables LLPS.***A*, liquid-like droplets formed of 50 μM Aβ_42_Os in 2 M ammonium chloride under different concentrations of SDS. Images were taken under fluorescence microscopy with 63× objective (oil immersion). *B*, secondary structure of Aβ_42_Os in 10 mM phosphate buffer, 10 mM NaCl, pH 7.4 under different concentrations of SDS. Data were shown as mean of five individual experiments. *C*, native-PAGE of 100 μM Aβ_42_Os under different concentrations of SDS. Lane 1: 20 μM monomeric Aβ_42_ peptide as control, lane 2: Aβ_42_Os formed without SDS, lane 3 to 7: Aβ_42_Os formed in 0.1% SDS (lane 3), 0.2% SDS (lane 4), 0.4% SDS (lane 5), 0.8% SDS (lane 6), 2% SDS (lane 7), lane 8: Albumin from chicken egg white (MW ∼ 44 kDa), and lane 9: Bovine Serum Albumin (MW ∼ 66 kDa). The *dash line* indicates the splice border of two native gels that ran in the same experiment. *D*, superposition of 1D ^1^H NMR spectra in aromatic and amide proton region of 200 μM Aβ_42_ monomeric polypeptides (*in black*), Aβ_42_Os formed in 0.2% (w/v) SDS (*in red*), and Aβ_42_Os formed in 0.2% SDS and contained 68 mM ammonium sulfate (*in blue*). All the experiments were recorded at 298 K using a Bruker Avance III 700 MHz spectrometer. LLPS, liquid–liquid phase separation; MW, molecular weight.

To examine whether the LLPS phenomenon of Aβ_42_Os is exclusively featured for the above mentioned Aβ_42_Os, another two methods for oligomers preparation were employed, including off-pathway Aβ-derived diffusible aggregates (24, 25) and on-pathway oligomers generated from low-salt, low-temperature condition (26). The oligomers induced by these two methods cannot undergo LLPS. Intriguingly, upon addition of SDS, liquid-like droplets rapidly formed of these two types of oligomers (Fig. S2). In a concentration range from 0.1% to 2% SDS, the droplet size increased as the concentration of SDS increased, and we propose the relatively large droplet formation is due to a faster fusion event (Fig. 2*A*). To further investigate the possible reason that SDS is essential to induce LLPS of Aβ_42_Os, we carried out CD and native-PAGE measurements to analyze the structural changes of Aβ_42_Os upon addition of SDS.

The on-pathway oligomers produced by low-salt, low-temperature condition are disc-shaped pentamers and adopt loosely aggregated strands with solvent accessible turns conformation. Upon addition of SDS, the pentamers break down into trimers and tetramers (26). In our study, Aβ_42_Os are composed of α-helix structure in the presence of SDS as shown by CD spectra (Fig. 2*B*). Besides, native-PAGE result showed Aβ_42_ formed a new assembly with higher MW of ∼ 66 kDa in the SDS concentration range (Fig. 2*C*). Altogether, these results indicate the potential role of secondary and tertiary structure induced by SDS environment in phase separation. We speculate that SDS reconfigures Aβ_42_Os to form a different MW assembly, which is required for protein LLPS. The remodulation may be due to the interactions between hydrophobic chains of SDS and hydrophobic residues in the C-terminal of Aβ_42_.

Additionally, NMR spectra were recorded to obtain information on protein folding and interactions in dilute phase. The dispersed 1D ^1^H spectrum of Aβ_42_ (black in Fig. 2*D*) indicates the unfolded, random coil conformation of monomeric peptides. Poor chemical shift dispersion and low signal intensity of Aβ_42_Os in 0.2% SDS (red in Fig. 2*D*) can be attributed to the coexistence of two-state folding and unfolding transitions. We speculate that there is a chemical exchange of unfolded form of monomer and well-folded form of oligomers. The fraction of protein in dilute phase contributes to the total NMR signal, whereas the fraction of protein in condensed phase causes line broadening leading to poor spectral resolution. Due to the presence of both dilute phase and condensed phase, further decrease of both NMR signal and spectral dispersion of Aβ_42_Os in phase-separated state (blue in Fig. 2*D*) were observed, reflecting that the occurrence of phase separation induces molecules coprecipitating inside the protein dense phase.

### LLPS of Aβ_42_Os depends on hydrophobic interactions

**Figure 3:**
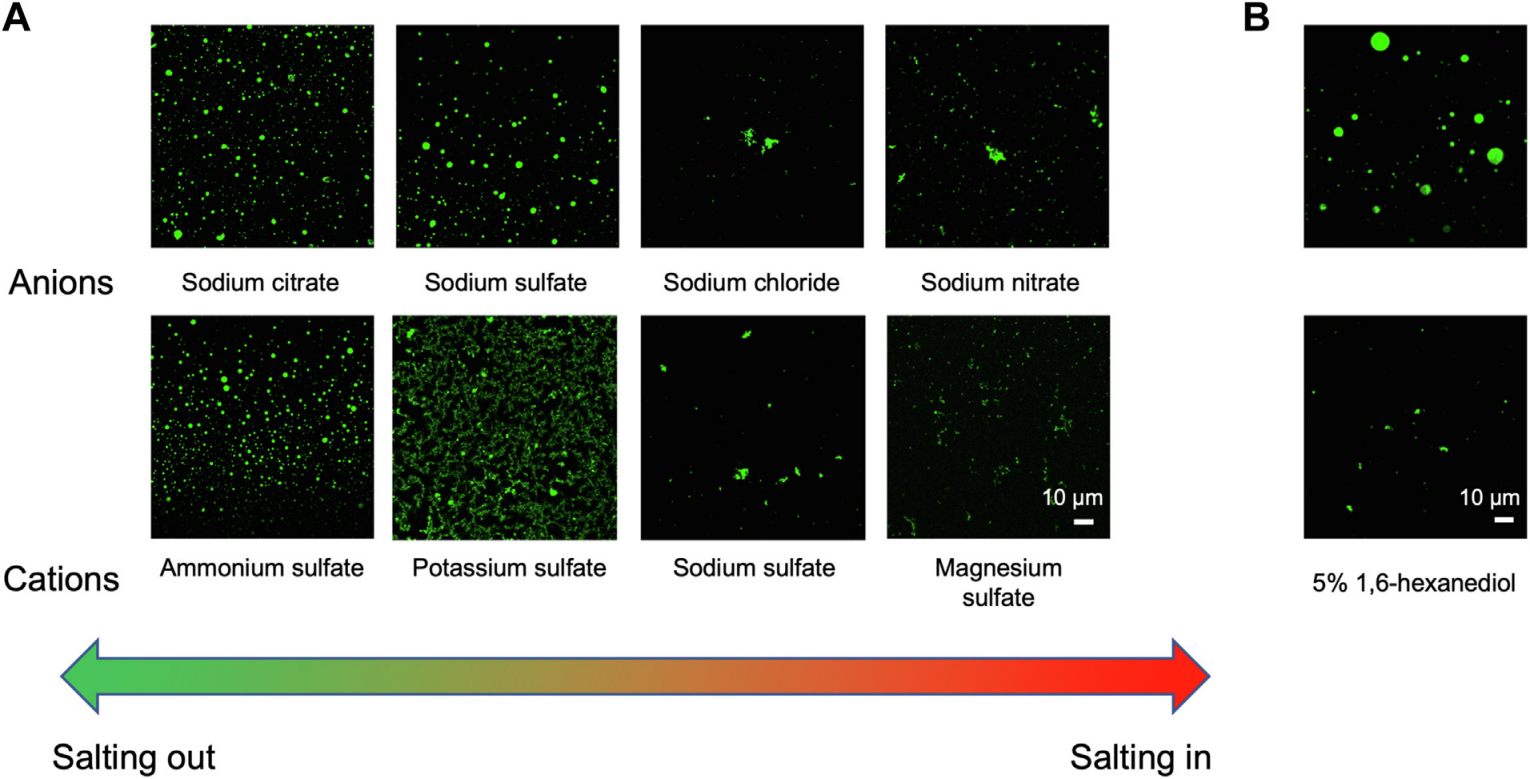
**LLPS of Aβ**_**42**_**Os depends on hydrophobic interactions.***A*, liquid-like droplet formed by 200 μM Aβ_42_Os under different ion conditions. The anions and cations we chose from left to right were subject to Hofmeister Series: citrate^3-^ > SO_4_^2-^ > Cl^-^ > NO_3_^-^, NH_4_^+^ > K^+^ > Na^+^ > Mg^2+^. *B*, liquid-like droplet formed of 200 μM Aβ_42_Os in the absence and presence of 5% (w/v) 1,6-hexanediol. LLPS, liquid–liquid phase separation.

Besides SDS, we further sought to find out the factors that influence on LLPS of Aβ_42_Os. The droplets formation of Aβ_42_Os was independent of temperature as similar droplets were observed at different temperatures (Fig. S3*A*). In addition, the pH conditions (range from pH 3.5 to pH 8.5) had negligible effects on its phase separation (Figs. 1*C* and S3*B*). Through systematical screenings, we found that liquid-like droplets formed in the presence of salts which were composed of strongly hydrated anions (such as citrate^3-^) and weakly hydrated cations (such as NH_4_^+^). In contrast, we observed the formation of aggregates in the presence of salts which were composed of weakly hydrated anions (such as NO_3_^-^) and strongly hydrated cations (such as Mg^2+^) (Fig. 3*A*). The results can be explained by Hofmeister phenomena (27). The Hofmeister Series shows the denaturation effectiveness of ions on proteins in sequences by interacting with water molecules, which thus enables or disables the formation of hydration shell. The formation of hydration shell decreases the number of free water molecules that interact with proteins, suggesting the hydrophobic interactions play a vital role in maintaining protein stability. More specifically, ions (such as NH_4_^+^ and citrate^3-^) exhibit strong salting-out ability and promote protein molecules interactions rather than protein–solvent interactions. In comparison with salts in the relative later positions of the Hofmeister Series (such as Mg^2+^ and NO_3_^-^), the solubility of proteins is increased by weakening the strength of hydrophobic interactions.

Moreover, upon addition of 5% (w/v) 1,6-hexanediol, the droplets were dissolved indicating the suppression of droplets formation (Fig. 3*B*). It further validates that Aβ_42_Os LLPS is promoted by hydrophobic interactions. These findings are in coincidence with previous studies on other intrinsically disordered proteins, suggesting that a general rule of hydrophobic interactions govern LLPS (28). As the presence of ions can modulate protein LLPS through regulating hydrophobic and electrostatic interactions, we explored how metal ions (Mg^2+^, Zn^2+^, and Al^3+^) influence on Aβ_42_Os LLPS. At the beginning of Aβ_42_Os LLPS, the addition of low concentration of both Mg^2+^ and Al^3+^ can stabilize the oligomers in its phase-separated state, whereas tiny protein aggregates were detected in the presence of Zn^2+^. It is noteworthy to mention that the liquid-like droplets formed in the presence of metal ions show slower fluidity which might indicate a change in the structural rigidity that are probably due to the interactions of metal ions and Aβ_42_Os. After coincubation quiescently of metal ions and phase-separated state of Aβ_42_Os at 37 °C for 24 h, bulk aggregates were observed in both cases, which suggests the retarding role of metal ions on Aβ_42_Os LLPS to form amyloid aggregates (Fig. S4) in comparison with the addition of metal ions directly into Aβ_42_ oligomeric state.

### LLPS of Aβ_42_Os redirects aggregation pathway and promotes amyloid formation

**Figure 4:**
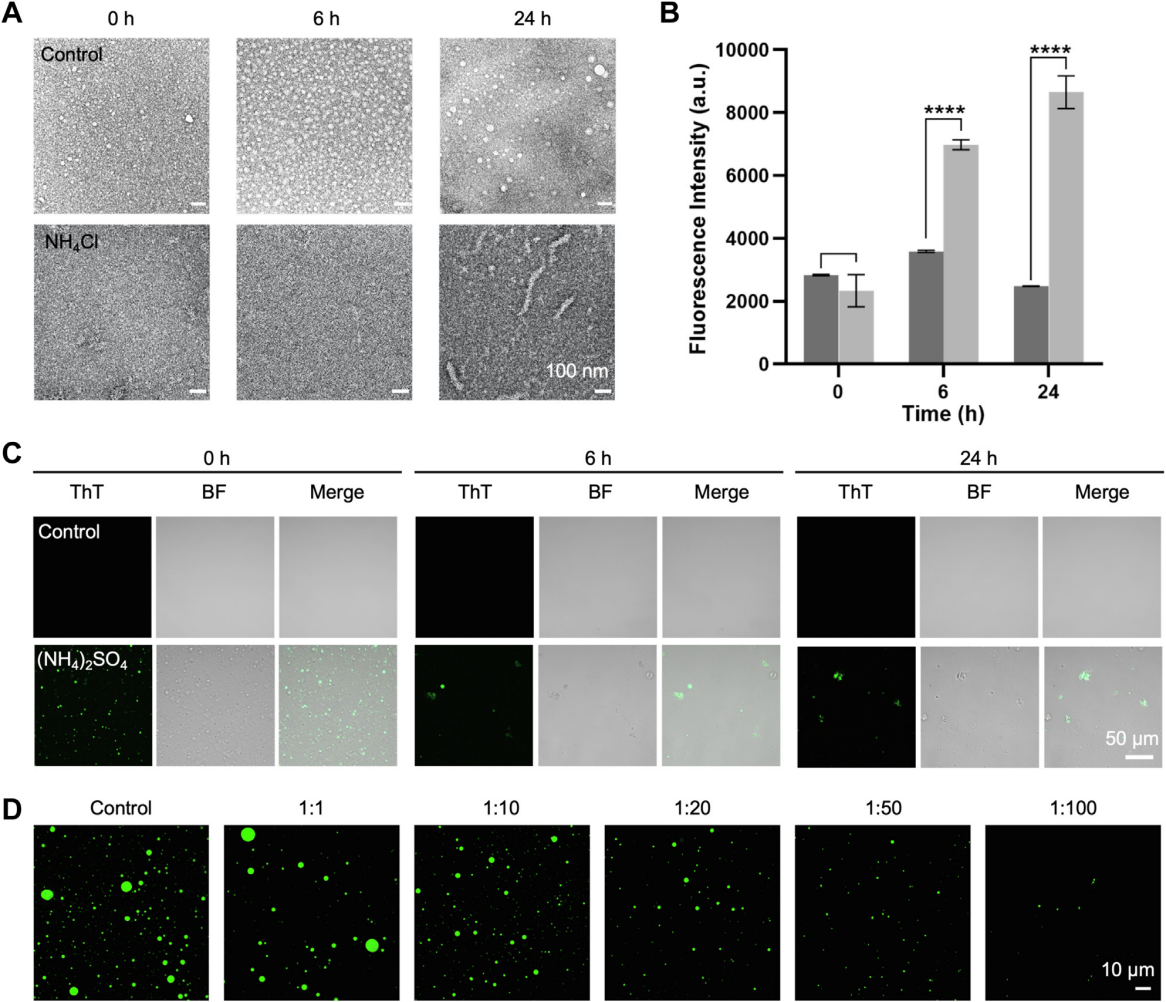
**LLPS of Aβ**_**42**_**Os redirects aggregation pathway and promotes amyloid formation.***A*, negative-staining TEM images of 200 μM Aβ_42_Os incubated in phase separation buffer condition including 10 mM phosphate buffer, 10 mM NaCl, 0.2% SDS, 0.02% NaN_3_, pH 7.4 containing 2 M ammonium chloride. Samples were incubated quiescently at 37 °C for 24 h. *B*, ThT fluorescent assay showed time-dependent amyloid fibrils formation of 200 μM Aβ_42_Os (prepared by the Protocol II) with (*in light gray*) and without (*in dark gray*) 2 M ammonium chloride. Samples were incubated quiescently at 37 °C for 24 h. Data were shown as means ± S.D., with n = 3 independent samples. *p* values were calculated using Student's *t* test. ∗∗∗∗*p* < 0.0001. *C*, confocal images of 200 μM Aβ_42_Os (prepared by the Protocol II) with and without 1 M ammonium sulfate incubated quiescently at 37 °C for 24 h. *D*, confocal images of 200 μM Aβ_42_Os droplets in the absence (control) and presence of EGCG under various substrate ratios. LLPS, liquid–liquid phase separation; TEM, transmission electron microscopy; EGCG, epigallocatechin gallate; ThT, thioflavin-T.

In addition to involving in phase separation, hydrophobic interactions are found to serve as the key driving force for amyloid fibrils formation (29). To investigate how Aβ_42_Os LLPS correlates with fibrillization, we incubated Aβ_42_Os in the phase separation buffer condition for days. As controls, we monitored phase separation and aggregation of Aβ_42_Os alone. SDS-trapped Aβ_42_ in its oligomeric form and almost no fibrillar structures were observed under TEM in 4 days (Fig. S5*A*). However, in the presence of either ammonium sulfate or ammonium chloride, fibrillar aggregates rapidly formed within 24 h (Figs. 4*A* and S5*A*). Meanwhile, thioflavin-T (ThT) assay was used to monitor and validate the presence of amyloid fibrils. The ThT fluorescence intensity showed gradual enhancement in the phase-separated state of Aβ_42_Os during 24 h incubation, which suggests the formation of amyloid fibrils adopting a cross-β structure. No significant ThT signal intensity increase was observed in the nonphase-separated state of Aβ_42_Os (Fig. 4*B*). In addition, ThT fluorescence showed large aggregates formation under light microscope. By contrast, Aβ_42_Os alone did not form any aggregates (Fig. 4*C*). The results imply that LLPS of Aβ_42_Os can redirect aggregation pathway into amyloid formation. The findings were confirmed by dot blot assay with anti-amyloid fibrils OC antibody (Fig. S5*B*). The chemiluminescence signal intensity of Aβ_42_Os which were incubated in ammonium sulfate solution condition remarkably increased in comparison with Aβ_42_Os alone, indicating an increase in the amount of amyloid fibrils.

We further used (−)-epigallocatechin gallate (EGCG) to investigate how amyloid inhibitor influences on the liquid droplet’s formation of Aβ_42_Os by applying different molar equivalent of EGCG to Aβ_42_Os. As the amount of EGCG increased, not only the number of liquid droplets became smaller but also the size of liquid droplets gradually shrank (Fig. 4*D*). At the ratio of 1:50 (peptide:EGCG), almost no droplets were observed under confocal microscope. Based on the previous research, EGCG could also bind with Aβ oligomers and alter their morphology to prevent the on-pathway fibrils formation (30, 31). Therefore, we suppose that EGCG enables to modulate LLPS of Aβ_42_Os by interacting with oligomers and alters the conformation of Aβ_42_Os, thus disfavoring LLPS.

## Discussion

Misfolded protein oligomerization, aggregation, and phase separation are critical biochemical processes linked to the pathogenesis and development of neurodegenerative diseases. Oligomerization is involved in phase separation of many proteins and is vital for biomolecular condensates formation (22, 32, 33). Recent study demonstrated that toxic tau oligomers were generated in RNA-mediated phase separation of tau protein, which presents the potential progression model of tau protein from monomeric state to high-order species (34). Amyloid oligomers are typically the most toxic species and responsible for the neurotoxicity in neurodegenerative diseases. Soluble AβOs assembled during aggregation exhibit high toxicity to cells even in the absence of mature Aβ fibrils, whereas Aβ deposition, one of the predominant pathological hallmarks of AD, do not always correlate well with disease occurrence and neuron disability (21, 35, 36). Thus, investigations into the relationship between protein oligomerization, aggregation, phase separation, and neurotoxicity are essential to our understanding of the pathobiology of neurodegenerative diseases.

Oligomerization can generate valency for protein LLPS and lower the energy barrier to demix the components into two distinct phases. Yet, the exact molecular mechanism for LLPS of oligomers is still unknown. Here, we reported AD-related Aβ_42_Os underwent LLPS *in vitro*. Sequence-based analysis (37) of Aβ_42_ shows low probability of occurrence of phase separation. The prediction outcome is consistent with experimental results that monomeric Aβ peptide showed no LLPS behavior under HiPPS profiling method (23). However, Aβ_42_Os rapidly underwent phase separation at a low concentration in the presence of SDS, proposing that SDS-induced oligomerization of higher MW assemblies may be a precursory process involved in protein LLPS. Liquid-like droplets were induced and formed under a buffer condition containing strong hydrated anions and weak hydrated cations based on Hofmeister Series. The specificity of AβOs rather than other species of Aβ undergoing LLPS in our findings may imply the pathological or biological relevance of LLPS in regard to the neurotoxicity in neurodegenerative diseases.

**Figure 5:**
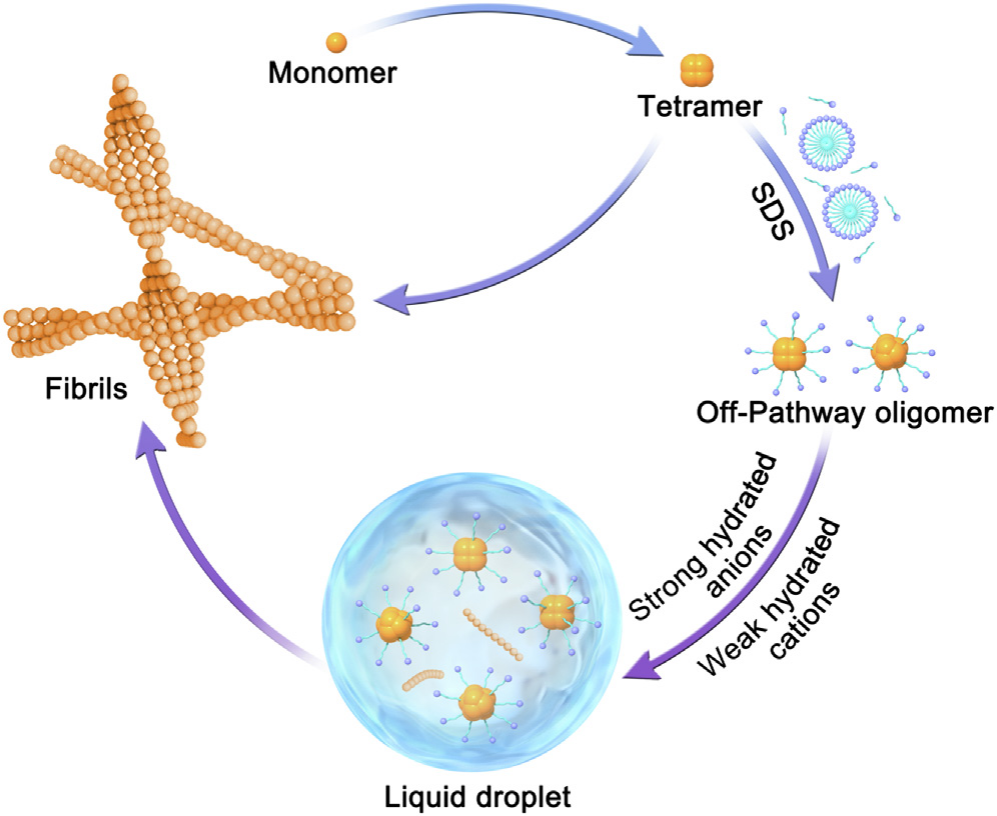
**LLPS in the landscape of Aβ aggregation.** Schematic shows that stable globular oligomers formed under the membrane-mimicking environment of SDS. Under a buffer condition containing strong hydrated anions and weak hydrated cations, LLPS can be observed and further to redirect aggregation pathway to form amyloid fibrils. LLPS, liquid–liquid phase separation.

Phase separation has been shown to be involved in fibrils formation in many cases such as islet amyloid polypeptide, tau, TDP-43 etc. (4, 7, 33). We also investigated the relationship between phase separation of Aβ_42_Os and amyloid fibrillization process. SDS is proposed to stabilize off-pathway oligomers, whereas Aβ_42_Os shift to on-pathway aggregation further to form amyloid fibrils while the phase separation occurs in our study (Fig. 5). We assume that LLPS induces a conformational change of Aβ_42_Os that favors fibrillization. The interactions may convert from sticker–spacer interactions that favor phase separation to cross-β interactions that favor fibrils formation. Several Aβ oligomers including both on- and off-pathway oligomers are characterized and exhibit different structures, including different loop and β-sheet alignment. Different molecular structures contain different underlying nucleation and aggregation pathways. It is of great interest to investigate the detailed structural information in the condensed phase of Aβ_42_Os.

It has been demonstrated that LLPS of FUS and TDP-43 can occur *via* hydrophobic and nonionic interactions under high-salt conditions and *via* hydrophilic interactions under low-salt conditions (28). In contrast, full-length α-synuclein cannot undergo instantaneous LLPS under low-salt conditions. Upon adding salts into the system, phase separation is triggered *via* hydrophobic interactions by non–amyloid-β component region of α-synuclein (38). We found that Aβ_42_Os rather than monomers functioned as the primary element to induce phase separation under high-salt conditions. As such, Aβ_42_Os show similar phase separation behaviors with these proteins, in which LLPS can be modulated by the presence of salts. These similarities suggest that hydrophobic interactions are involved in the phase separation of these proteins. The fact that Aβ_42_ only phase separates at higher-order oligomerization state under high-salt conditions indicates the driving forces governing phase separation are not only determined by protein sequence but also crucially sensitive to solution environment and oligomerization state.

Although Aβ_42_Os globulomer was prepared *in vitro* in our study, it is present in the AD patients’ brain and Aβ_42_-overproducing transgenic mice (24). Furthermore, hippocampal neurons can bind Aβ_42_Os in a specific punctuated manner and long-term potentiation is inhibited completely by this oligomer. Based on this research, Aβ_42_Os were formed in globulomer pathway independently of fibrillar aggregation pathway. The Aβ_42_Os globulomer has no tendency to aggregate into amyloid fibrils (24). However, phase separation of Aβ_42_Os globulomer redirects the aggregation to fibrillar aggregation pathway, which may be relevant to the pathological process of AD. In addition, our finding showed EGCG-retarded amyloid protein aggregation also inhibited Aβ_42_Os LLPS, which suggests that LLPS might be a critical step to nucleate its aggregation.

The physiological relevance of our finding and the direct *in vivo* implication of Aβ_42_Os LLPS in AD as AD-associated factors, which can accelerate Aβ aggregation, promote liquid-to-solid transitions and further form pathological assemblies of AD. Although how phase separation is related with amyloid formation is largely unknown, our findings provide the evidence that protein can be rearranged in the phase-separated state and targeting phase separation to block phase transition and protein aggregation can be a possible treatment strategy for neurodegenerative diseases. Aβ_42_Os have been shown to bind with various receptors on cell surface and thus induced downstream neurotoxicity. Aβ_42_Os complexed with cellular prion protein yielded a reversible hydrogel, which can trap signal-transducing mGluR5 at the cell surface and is present in human AD brain (39). The cophase separation of cellular prion protein and Aβ_42_Os is highly relevant to neurotoxicity, leading to synaptic dysfunction and loss. To study the localization of Aβ_42_Os’ liquid-like droplets in cells, we cultured BV-2 microglial cells and HT22 hippocampal neuronal cells with preformed Aβ_42_Os. The confocal images (Fig. S5*C*) showed the diffused localization of Aβ_42_Os in both cells, whereas condensed puncta can be observed upon addition of low concentration of ammonium sulfate. The cellular findings confirm the droplets formation of Aβ_42_Os either on the cell surface or inside the cells, suggesting the possible mechanism of phase separation involved in the downstream signaling.

In summary, we reported AD-related Aβ_42_Os underwent LLPS *in vitro* and this phase separation process can modulate amyloid fibrillization which is relevant to AD pathogenesis. The promoter role of oligomerization in phase separation is the key to understand the potential correlation of these early events involved in protein aggregation process, further to explain the lack of underlying mechanism of aberrant liquid-to-solid phase transitions in the pathogenesis of AD. In combination with the description of the relationship between phase separation and aggregation process, our findings provide an important insight into the complex mechanism of aberrant phase transition and aggregation of Aβ to better understand the pathogenesis and development of AD.

## Experimental procedures

### Sample preparation

Lyophilized synthetic Aβ_42_ peptide powder was dissolved in precooling hexafluoroisopropanol (HFIP) solvent to a final concentration of 1 mM and followed by water bath sonication for 10 min. Treated peptide was incubated at room temperature for 1 h to remove preformed aggregates. HFIP was evaporated overnight in a fume hood and Aβ_42_ peptide film was generated afterward. The peptide film can be stored at −80 °C for further usage.

#### Protocol I

The peptide film was resuspended in DMSO solvent to a final concentration of 5 mM by water bath sonication. The peptide solution was diluted into defined concentrations with 20 mM NaH_2_PO_4_, 140 mM NaCl aqueous buffer, pH 7.4 containing indicated concentrations of SDS. After 10 min centrifugation at the speed of 14,000 *g* at 4 °C, the supernatant was filtered through a 0.22 μm PVDF membrane to remove any insoluble aggregates. Then the peptide stock was prepared for further use.

#### Protocol II

HFIP-treated peptide film was dissolved in 100 mM NaOH aqueous solution to a final concentration of 20 mg/ml. Subsequently, the dissolved peptide solution was diluted with 10 mM phosphate buffer, 10 mM NaCl, pH 7.4 aqueous buffer containing indicated concentrations of SDS to defined peptide concentrations. After 10 min centrifugation at the speed of 14,000 g at 4 °C, the supernatant was filtered through a 0.22 μm PVDF membrane to remove any potential aggregates. Then the peptide stock was prepared for further use. Otherwise noted by the buffer composition, Aβ_42_Os for related experiments were prepared by the Protocol I.

The recombinant peptide was described in detail previously (40). Briefly, the plasmid construct of Aβ_42_ was expressed in BL21(DE3)pLysS *Escherichia coli* strains. Aβ_42_ peptides were purified by batch ion-exchange chromatography with diethylaminoethyl (DEAE) cellulose resin. The pure elution was desalted into 50 mM ammonium acetate pH 8.5 and subsequently lyophilized and stored in a freezer at −20 °C. The lyophilized recombinant Aβ_42_ powder followed the above sample preparation for further use.

### Transmission electron microscopy

Six microliters of peptide stock with indicated concentration were spotted on the carbon-coated formvar grid and incubated for 45 s. The grid was then dried with filter paper, washed with ultrapure water, and then stained with 3% (w/v) aqueous uranyl formate solution. The samples were dried before imaging and viewed with Thermo Fisher Scientific Talos F200C 200 kV transmission electron microscope and FEI Tecnai G2 Spirit BioTWIN 120 kV transmission electron microscope.

### Liquid–liquid phase separation

To study LLPS, 3 μl peptide stock was mixed with 3 μl indicated buffers and dropped onto a glass bottom dish equipped with a Zeiss 880 confocal microscopy, and the formation of droplets was observed in a bright field. For fluorescence microscopy imaging, unlabeled Aβ_42_Os was mixed with FITC-labeled Aβ_42_Os at the molar ratio of 25:1. Three microliters sample mixed with 3 μl indicated buffers were placed onto a glass bottom dish. Images were taken with a 63× objective (oil immersion).

To test the fluidity of the droplet, unlabeled Aβ_42_Os was mixed with FITC-labeled Aβ_42_Os at the molar ratio of 25:1 for fluorescence imaging to observe the droplet fusion process. After the droplet formation, a suitable area was selected for continuous photography until the droplet fusion. Images were taken with a 63× objective (oil immersion).

To investigate the effect of metal ions on Aβ_42_Os LLPS, 50 μM phase-separated state Aβ_42_Os under 1 M ammonium sulfate buffer condition were mixed with metal ion stock solutions including MgSO_4_, AlCl_3,_ and (CH_3_COO)_2_Zn, respectively. Metal ion stock solutions with 2 M were prepared by dissolving corresponding powder in ultrapure water. The final concentration of metal ions in the above sample mixture was 0.05 M. For fluorescence microscopy imaging, unlabeled Aβ_42_Os was mixed with FITC-labeled Aβ_42_Os at the molar ratio of 50:1. Six microliters of the above mixture samples were placed on the glass bottom dish and images were taken with Zeiss 880 confocal microscopy.

For the regulatory role of EGCG on LLPS, 20 mM EGCG stock solution was prepared by dissolving EGCG (Sigma-Aldrich) powder in ultrapure water. Aβ_42_Os alone or in a mixture with EGCG at different molar ratios were prepared for droplets formation observation. At the indicated molar ratios, aliquots were gently mixed with ammonium chloride solution to a final concentration of 200 μM Aβ_42_Os and 2 M ammonium chloride. Six microliters of the above mixture sample were placed on the glass bottom dish and images were taken with Zeiss 880 confocal microscopy.

To study Aβ_42_Os LLPS in cells, we cultured two different cell lines including BV-2 microglial cells and HT22 hippocampal neuronal cells. Cells were mixed with 1 ml Dulbecco′s Modified Eagle′s Medium, then transferred into a confocal dish with 100,000 cells per dish and cultured overnight in a CO_2_ incubator. The peptide stock was incubated at 37 °C for 6 h and then added into the cells to a final concentration of 10 μM. Unlabeled Aβ_42_Os was mixed with FITC-labeled Aβ_42_Os at the molar ratio of 10:1 for fluorescence imaging. The cells were treated with Aβ_42_Os for 2 h and detected by a confocal laser microscope immediately.

### Fluorescence recovery after photobleaching

All the reaction mixtures contained FITC-labeled Aβ_42_Os and unlabeled Aβ_42_Os with a molar ratio 1:25 to minimize the effect of protein labeling on LLPS. The solution was spotted onto a 15 mm glass bottom dish. After droplet formation was confirmed by fluorescence microscopy, the dish containing the droplets were subjected to FRAP experiments at various time points as indicated in the respective sections. Photobleaching of the phase-separated droplets was carried out using a 488 nm laser. Intensity was recorded from three different region of interests (ROIs) of constant radii: actual bleaching region (ROI-1), reference region on the nearby droplet (ROI-2) to correct for passive bleaching during laser exposure, and a region outside the droplet to correct for background fluorescence intensity (ROI-3). FRAP studies were performed using a laser scanning confocal microscope (Zeiss 880) with a 63× objective (oil immersion). The selected ROI-1 was bleached with 100% laser power. All the measurements were performed at room temperature and for five individual droplets.

### NMR measurements

To exclude the interference of DMSO, we prepared Aβ_42_Os NMR samples by the Protocol II. Peptide film was dissolved in 100 mM NaOH aqueous solution and diluted with 10 mM phosphate buffer, 10 mM NaCl, pH 7.4 containing 0.2% SDS to a final concentration of 200 μM Aβ_42_Os. Three NMR samples including 200 μM Aβ_42_ monomeric peptides only, 200 μM Aβ_42_Os in 10 mM phosphate buffer, 10 mM NaCl, pH 7.4 containing 0.2% (w/v) SDS, and 200 μM Aβ_42_Os in 0.2% (w/v) SDS mixed with phase separation buffer ammonium sulfate were performed at the temperature of 298 K using 64 scans and 32 k time domain points. All the 1D proton NMR spectra were carried out on a Bruker Avance III 700 MHz spectrometer, equipped with a triple resonance cryo-probe. The experimental data were processed with TopSpin 4.1.3.

### CD spectroscopy

For oligomers produced by the Protocol I, 50 μM peptide stock solution was firstly exchanged into 5 mM NaH_2_PO_4_, 35 mM NaCl, pH 7.4 aqueous buffer containing 0.2% SDS by using 10 kDa cut-off spin filter. Afterward, 400 μl volume of 50 μM Aβ_42_Os was transferred to a CD micro cuvette of 0.1 cm path length for measurement. Concerning oligomers produced by the Protocol II, 400 μl volume of 50 μM Aβ_42_Os in 10 mM phosphate buffer, 10 mM NaCl, pH 7.4 containing indicated concentration of SDS was transferred to a CD micro cuvette for measurement. CD measurements were conducted by using AVIV Model 420SF spectrophotometer and buffer was always subtracted from CD spectra of all the peptide solutions. Spectra were recorded from 260 to 190 nm with a speed of 100 nm/min at 25 °C and each spectrum was the average of five spectra.

### Native-PAGE

Aβ_42_Os with indicated concentration of SDS were mixed with sample loading buffer and loaded onto 17% native gels for electrophoresis at 120 V for 2.5 h in ice bath. The gel was visualized with Coomassie brilliant blue stain. Bovine serum albumin (MW ∼ 66 kDa) and albumin from chicken egg white (MW ∼ 44 kDa) were used as protein ladders to estimate MW of Aβ_42_Os.

### ThT assay

ThT, as a fluorescent marker for amyloid deposits, is applied to monitor and quantify the presence of misfolded aggregates and amyloid fibrils *in vitro*. Two hundred fifty microliters peptide stock were mixed with 250 μl ammonium chloride solution (at the ratio of 1/1) to a final concentration of 200 μM peptide and 2 M ammonium chloride. The mixture sample was incubated quiescently at 37 °C for ThT assay. At the indicated time points, 50 μl aliquots were gently mixed with 50 μl of 200 μM ThT in 0.05 M glycine/NaOH, pH 8.5 at room temperature. ThT fluorescence intensity was determined immediately by measuring fluorescence emission at 485 nm following excitation at 440 nm with Molecular Device SpectraMax i3x.

### Dot blot assay

A nitrocellulose membrane was divided into equal grids, and 2 μl of 200 μM peptide stock sample was dotted onto the membrane and then dried with air flow. The membrane was blocked in Tris buffered saline with Tween-20 (TBST) solution (10 mM Tris–HCl, 150 mM NaCl, and 0.1% (v/v) Tween-20) containing 10% milk at 4 °C overnight. Subsequently, the membrane was incubated with anti-amyloid fibrils OC antibody (1:1000) or anti-oligomer A11 antibody (1:1000) at 4 °C with gently shaking overnight. Following three times of wash with TBST, the membrane was incubated with secondary antibody (Goat Anti-Rabbit IgG, 1:5000) at 25 °C for 2 h. Blots were washed three times with TBST and each time for 10 min and then developed with Omni-ECL Femto Light Chemiluminescence kit.

## Data availability

All data presented are contained within the manuscript.

## Supporting information

This article contains supporting information (supplementary Figs. S1–S5) (24, 25, 26).

## Conflict of interest

The authors declare that they have no conflicts of interest with the contents of this article.

## References

[1] Chiti F., Dobson C.M. (2006). Protein misfolding, functional amyloid, and human disease. Annu. Rev. Biochem..

[2] Chiti F., Dobson C.M. (2017). Protein misfolding, amyloid formation, and human disease: a summary of progress over the last decade. Annu. Rev. Biochem..

[3] Iadanza M.G., Jackson M.P., Hewitt E.W., Ranson N.A., Radford S.E. (2018). A new era for understanding amyloid structures and disease. Nat. Rev. Mol. Cell Biol..

[4] Wegmann S., Eftekharzadeh B., Tepper K., Zoltowska K.M., Bennett R.E., Dujardin S. (2018). Tau protein liquid–liquid phase separation can initiate tau aggregation. EMBO J..

[5] Kanaan N.M., Hamel C., Grabinski T., Combs B. (2020). Liquid-liquid phase separation induces pathogenic tau conformations *in vitro*. Nat. Commun..

[6] Ray S., Singh N., Kumar R., Patel K., Pandey S., Datta D. (2020). α-Synuclein aggregation nucleates through liquid–liquid phase separation. Nat. Chem..

[7] Pytowski L., Lee C.F., Foley A.C., Vaux D.J., Jean L. (2020). Liquid–liquid phase separation of type II diabetes-associated IAPP initiates hydrogelation and aggregation. Proc. Natl. Acad. Sci. U. S. A..

[8] Hernández-Vega A., Braun M., Scharrel L., Jahnel M., Wegmann S., Hyman B.T. (2017). Local nucleation of microtubule bundles through tubulin concentration into a condensed tau phase. Cell Rep..

[9] Molliex A., Temirov J., Lee J., Coughlin M., Kanagaraj A.P., Kim H.J. (2015). Phase separation by low complexity domains promotes stress granule assembly and drives pathological fibrillization. Cell.

[10] Zbinden A., Pérez-Berlanga M., De Rossi P., Polymenidou M. (2020). Phase separation and neurodegenerative diseases: a disturbance in the force. Dev. Cell..

[11] Patel A., Lee H.O., Jawerth L., Maharana S., Jahnel M., Hein M.Y. (2015). A liquid-to-solid phase transition of the ALS protein FUS accelerated by disease mutation. Cell.

[12] Brangwynne C.P., Tompa P., Pappu R.V. (2015). Polymer physics of intracellular phase transitions. Nat. Phys..

[13] Dignon G.L., Best R.B., Mittal J. (2020). Biomolecular phase separation: from Molecular Driving forces to macroscopic properties. Annu. Rev. Phys. Chem..

[14] Vernon R.M., Chong P.A., Tsang B., Kim T.H., Bah A., Farber P. (2018). Pi-Pi contacts are an overlooked protein feature relevant to phase separation. eLife.

[15] Murthy A.C., Dignon G.L., Kan Y., Zerze G.H., Parekh S.H., Mittal J. (2019). Molecular interactions underlying liquid−liquid phase separation of the FUS low-complexity domain. Nat. Struct. Mol. Biol..

[16] Gui X., Luo F., Li Y., Zhou H., Qin Z., Liu Z. (2019). Structural basis for reversible amyloids of hnRNPA1 elucidates their role in stress granule assembly. Nat. Commun..

[17] Wen J., Hong L., Krainer G., Yao Q.-Q., Knowles T.P.J., Wu S. (2021). Conformational expansion of tau in condensates promotes irreversible aggregation. J. Am. Chem. Soc..

[18] Burke K.A., Janke A.M., Rhine C.L., Fawzi N.L. (2015). Residue-by-Residue view of *in vitro* FUS granules that bind the C-terminal domain of RNA polymerase II. Mol. Cell.

[19] Lin Y., Protter D.S.W., Rosen M.K., Parker R. (2015). Formation and maturation of phase-separated liquid droplets by RNA-binding proteins. Mol. Cell.

[20] Alberti S., Dormann D. (2019). Liquid–liquid phase separation in disease. Annu. Rev. Genet..

[21] Lee S.J.C., Nam E., Lee H.J., Savelieff M.G., Lim M.H. (2017). Towards an understanding of amyloid-β oligomers: characterization, toxicity mechanisms, and inhibitors. Chem. Soc. Rev..

[22] Wang A., Conicella A.E., Schmidt H.B., Martin E.W., Rhoads S.N., Reeb A.N. (2018). A single N-terminal phosphomimic disrupts TDP-43 polymerization, phase separation, and RNA splicing. EMBO J..

[23] Li Y., Gu J., Liu C., Li D. (2022). A high-throughput method for exploring the parameter space of protein liquid-liquid phase separation. Cell Rep. Phys. Sci..

[24] Barghorn S., Nimmrich V., Striebinger A., Krantz C., Keller P., Janson B. (2005). Globular amyloid beta-peptide1-42 oligomer - a homogenous and stable neuropathological protein in Alzheimer’s disease. J. Neurochem..

[25] Dahlgren K.N., Manelli A.M., Stine W.B., Baker L.K., Krafft G.A., LaDu M.J. (2002). Oligomeric and fibrillar species of amyloid-β peptides differentially affect neuronal viability. J. Biol. Chem..

[26] Ahmed M., Davis J., Aucoin D., Sato T., Ahuja S., Aimoto S. (2010). Structural conversion of neurotoxic amyloid-β1–42 oligomers to fibrils. Nat. Struct. Mol. Biol..

[27] Cacace M.G., Landau E.M., Ramsden J.J. (1997). The hofmeister series: salt and solvent effects on interfacial phenomena. Q. Rev. Biophys..

[28] Krainer G., Welsh T.J., Joseph J.A., Espinosa J.R., Wittmann S., de Csilléry E. (2021). Reentrant liquid condensate phase of proteins is stabilized by hydrophobic and non-ionic interactions. Nat. Commun..

[29] Sawaya M.R., Hughes M.P., Rodriguez J.A., Riek R., Eisenberg D.S. (2021). The expanding amyloid family: structure, stability, function, and pathogenesis. Cell.

[30] Ehrnhoefer D.E., Bieschke J., Boeddrich A., Herbst M., Masino L., Lurz R. (2008). EGCG redirects amyloidogenic polypeptides into unstructured, off-pathway oligomers. Nat. Struct. Mol. Biol..

[31] Ahmed R., VanSchouwen B., Jafari N., Ni X., Ortega J., Melacini G. (2017). Molecular mechanism for the (−)-Epigallocatechin gallate-induced toxic to nontoxic remodeling of Aβ oligomers. J. Am. Chem. Soc..

[32] Marzahn M.R., Marada S., Lee J., Nourse A., Kenrick S., Zhao H. (2016). Higher-order oligomerization promotes localization of SPOP to liquid nuclear speckles. EMBO J..

[33] Babinchak W.M., Haider R., Dumm B.K., Sarkar P., Surewicz K., Choi J.-K. (2019). The role of liquid–liquid phase separation in aggregation of the TDP-43 low-complexity domain. J. Biol. Chem..

[34] Ash P.E.A., Lei S., Shattuck J., Boudeau S., Carlomagno Y., Medalla M. (2021). TIA1 potentiates tau phase separation and promotes generation of toxic oligomeric tau. Proc. Natl. Acad. Sci. U. S. A..

[35] Glabe C.G. (2008). Structural classification of toxic amyloid oligomers. J. Biol. Chem..

[36] Benilova I., Karran E., De Strooper B. (2012). The toxic Aβ oligomer and Alzheimer’s disease: an emperor in need of clothes. Nat. Neurosci..

[37] Chu X., Sun T., Li Q., Xu Y., Zhang Z., Lai L. (2022). Prediction of liquid–liquid phase separating proteins using machine learning. BMC Bioinform..

[38] Sawner A.S., Ray S., Yadav P., Mukherjee S., Panigrahi R., Poudyal M. (2021). Modulating α-synuclein liquid–liquid phase separation: published as part of the *biochemistry* virtual special issue “protein condensates. Biochemistry.

[39] Kostylev M.A., Tuttle M.D., Lee S., Klein L.E., Takahashi H., Cox T.O. (2018). Liquid and hydrogel phases of PrPC linked to conformation shifts and triggered by Alzheimer’s amyloid-β oligomers. Mol. Cell..

[40] Walsh D.M., Thulin E., Minogue A.M., Gustavsson N., Pang E., Teplow D.B. (2009). A facile method for expression and purification of the Alzheimer’s disease-associated amyloid β-peptide. FEBS J..

