# Supplementary material for: Liquid–liquid phase separation of amyloid-β oligomers modulates amyloid fibrils formation
Source: J Biol Chem. 2023 Jan 20;299(3):102926. doi: 10.1016/j.jbc.2023.102926 (PMC9974441; doi:10.1016/j.jbc.2023.102926)
Supplement: Supporting information [file mmc1.docx]

**Liquid–liquid phase separation of amyloid-β oligomers modulates amyloid fibrils formation**

Xinrui Gui^1,‡^, Shuang Feng^2,‡^, Zilong Li^1,‡^, Yanyan Li^2^, Bernd Reif^3,4,^*, Bingyang Shi^1,5,^*, and Zheng Niu^2,3,^*

1. Henan-Macquarie University Joint Centre for Biomedical Innovation, School of Life Sciences, Henan University, Kaifeng, China.

2. Henan Key Laboratory of Brain Targeted Bio-nanomedicine, School of Pharmacy, Henan University, Kaifeng, China.

3. Munich Center for Integrated Protein Science (CIPS-M) at the Department of Chemistry, Technische Universität München (TUM), Garching, Germany.

4. Deutsches Forschungszentrum für Gesundheit und Umwelt Institute of Structural Biology, Helmholtz-Zentrum München (HMGU), Neuherberg, Germany.

5. Macquarie Medical School, Faculty of Medicine & Health Sciences, Macquarie University, Sydney, NSW, Australia

^‡^ These authors contributed equally to this work.

* For correspondence: Bernd Reif, reif@tum.de; Bingyang Shi, bs@henu.edu.cn;

Zheng Niu, nz@henu.edu.cn.

**Supplementary figures**


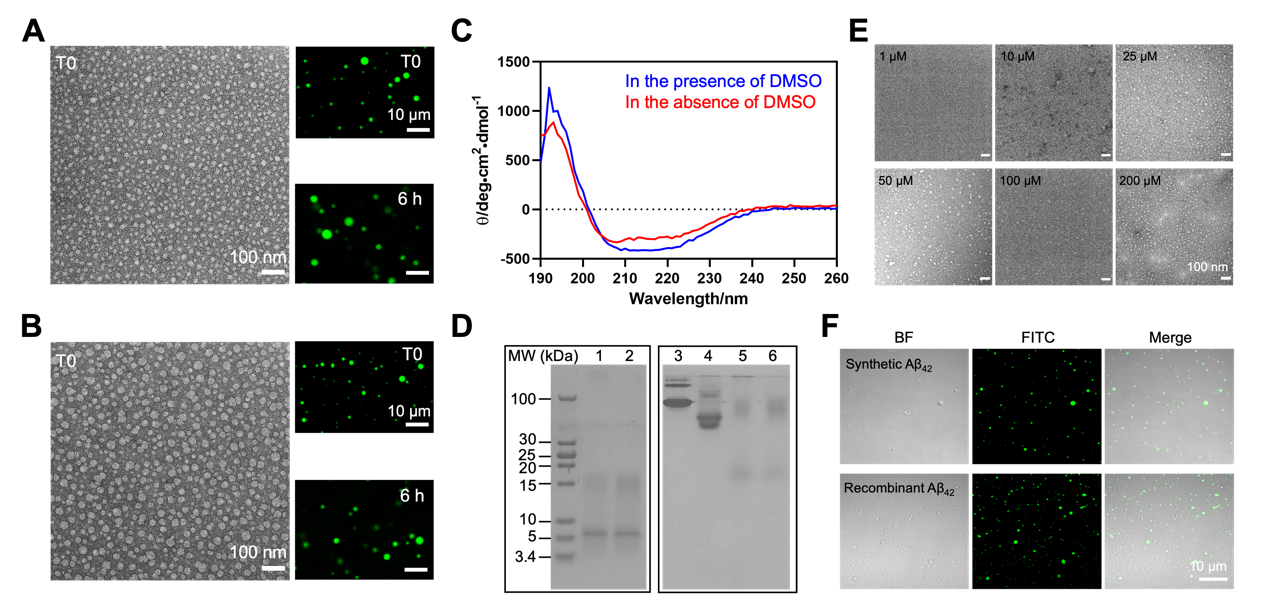


**Figure S1**: To evaluate whether the two different oligomers preparation protocols produce similar Aβ_42_Os, we performed TEM imaging technique, CD measurements and protein electrophoresis. The Aβ_42_Os generated by these two protocols are comparable. A) TEM images of 100 µM Aβ_42_Os prepared by the Protocol I. Spherical oligomers were observed from TEM. Liquid-like droplets were formed under 1 M ammonium sulfate at T0 and 6 h incubation at 37°C quiescently. B) TEM images of 100 µM Aβ_42_Os prepared by the Protocol II. In comparison with the oligomers produced by the Protocol I, similar spherical oligomers were observed. Liquid-like droplets were formed under 1 M ammonium sulfate at T0 and 6 h incubation at 37°C quiescently. C). Secondary structure measurements of 50 µM Aβ_42_Os produced either by the Protocol I (in blue) or by the Protocol II (in red). Data were shown as mean of 5 individual experiments. D) Tricine-SDS-PAGE and native-PAGE of 100 µM Aβ_42_Os were prepared from both protocols. Lane1 and lane 5: 100 µM Aβ_42_Os prepared by the Protocol II, lane 2 and lane 6: 100 µM Aβ_42_Os prepared by the Protocol I, lane 3: Bovine Serum Albumin (MW ~ 66 kDa), lane 4: Albumin from chicken egg white (MW ~ 44 kDa). The bands with MW of ~18 kDa in Tricine-SDS-PAGE and higher MW of ~66 kDa in native-PAGE were demonstrated the MW of these two oligomers are comparable. E) TEM images of Aβ_42_Os with different concentrations. TEM image taken from 200µM Aβ_42_Os is identical to Figure 1A. F) Confocal images of droplets formed by 200 µM Aβ_42_Os which were prepared from both synthetic and recombinant Aβ_42_ peptide in 10 mM phosphate buffer, 10 mM NaCl, pH 7.4 containing 0.2% (w/v) SDS and 1 M ammonium sulfate.


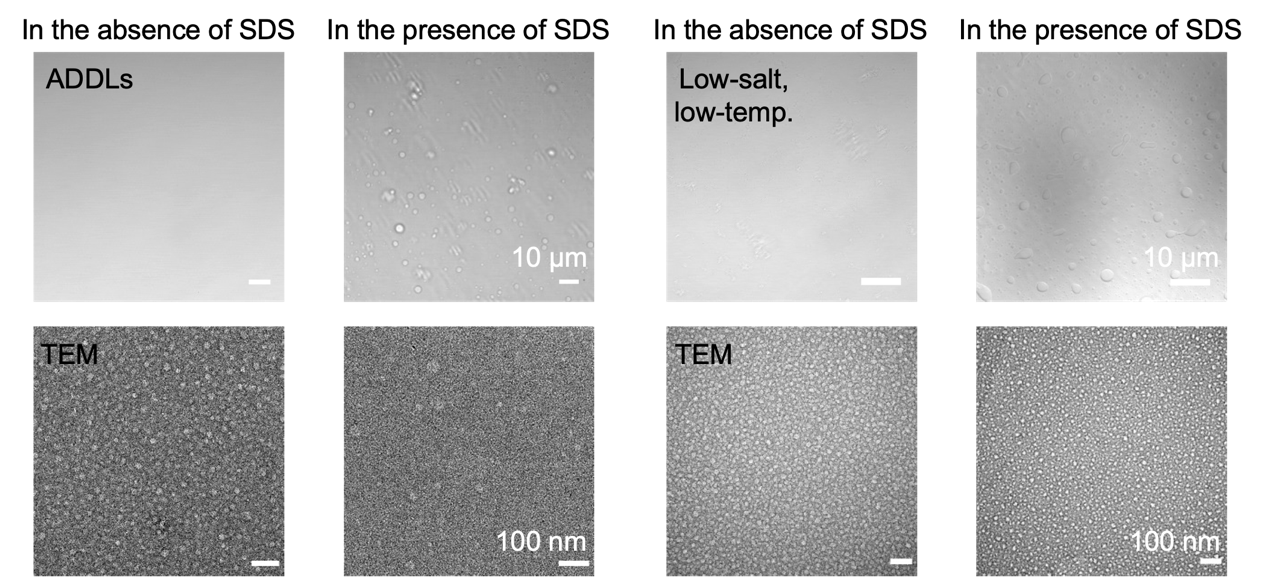


**Figure S2**: Confocal and TEM images of both Aβ-derived diffusible aggregates (ADDLs) and oligomers prepared by low-salt and low-temperature condition in the absence and presence of 0.2% (w/v) SDS. ADDLs (24, 25) and low-salt, low-temperature oligomers (26) preparation details were described previously. For ADDLs, TEM images were taken from 100 µM monomeric Aβ_42_ peptides that were incubated at 4°C for 24 h in DMEM-F12 medium with and without SDS. For low-salt and low temperature oligomers, TEM images were taken from 100 µM Aβ_42_ oligomers that were prepared in 10 mM phosphate buffer, 10 mM NaCl, pH 7.4 with and without SDS. The details for confocal images are listed as below:

| Preparation | Oligomers | HiPPS profiling method condition |
| --- | --- | --- |
| ADDLs | 50 µM Aβ_42_ oligomers in DMEM-F12 medium | 2 M ammonium chloride with and without 0.2% (w/v) SDS |
| Low-salt, low-temp. | 200 µM Aβ_42_ oligomers formed in 10 mM phosphate buffer, 10 mM NaCl, pH 7.4 | 1 M ammonium sulfate with and without 0.2% (w/v) SDS |


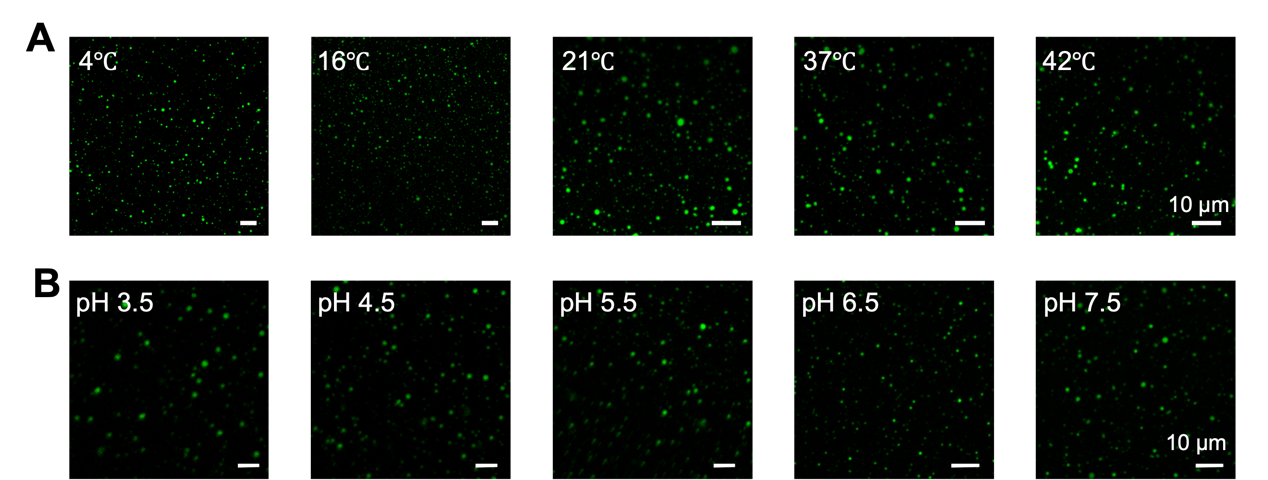


**Figure S3**: Droplets formation of Aβ_42_Os under various temperature and pH conditions. Similar phase separation phenomena were observed at various temperature and different pH conditions. HiPPS profiling method conditions are listed as below:

| 4°C ~ 42°C | 200 µM Aβ_42_Os in 1 M ammonium sulfate, 0.05 M Tris-HCl buffer, pH 8.5 |
| --- | --- |
| pH 3.5 | 200 µM Aβ_42_Os in 1 M ammonium sulfate, 0.05 M citric acid buffer, pH 3.5 |
| pH 4.5 | 200 µM Aβ_42_Os in 1 M ammonium sulfate, 0.05 M citric acid buffer, pH 4.5 |
| pH 5.5 | 200 µM Aβ_42_Os in 1 M ammonium sulfate, 0.05 M Bis-Tris buffer, pH 5.5 |
| pH 6.5 | 200 µM Aβ_42_Os in 1 M ammonium sulfate, 0.05 M Bis-Tris buffer, pH 6.5 |
| pH 7.5 | 200 µM Aβ_42_Os in 1 M ammonium sulfate, 0.05 M HEPES buffer, pH 7.5 |


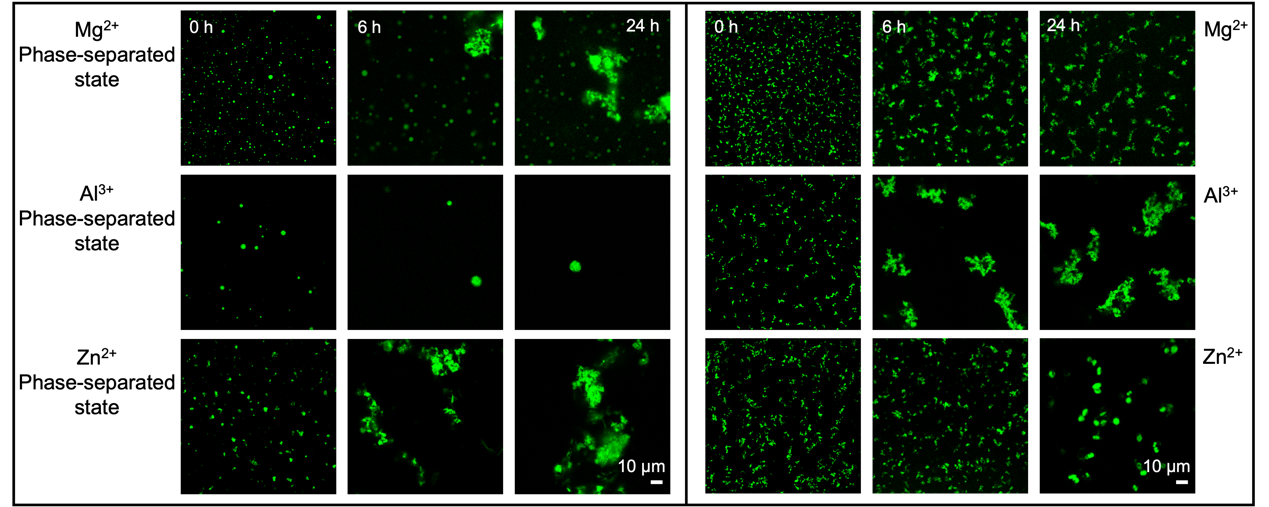


**Figure S4**: Metal ions including Mg^2+^, Zn^2+^ and Al^3+^ influence on Aβ_42_Os LLPS. Confocal images of phase-separated state Aβ_42_Os (50 µM Aβ_42_Os in 20 mM NaH_2_PO_4_, 140 mM NaCl, pH 7.4 aqueous buffer containing 0.2% SDS and 1 M ammonium sulfate) in the presence of 0.05 M MgSO_4_, AlCl_3_ and (CH_3_COO)_2_Zn, respectively. After sample preparation, the phase-separated state Aβ_42_Os with metal ions were incubated immediately at 37°C for 24 h quiescently, and the corresponding confocal images were recorded at different time intervals. As controls, Aβ_42_Os alone in the presence of Mg^2+^, Zn^2+^ and Al^3+^ were incubated at 37°C for 24 h quiescently.


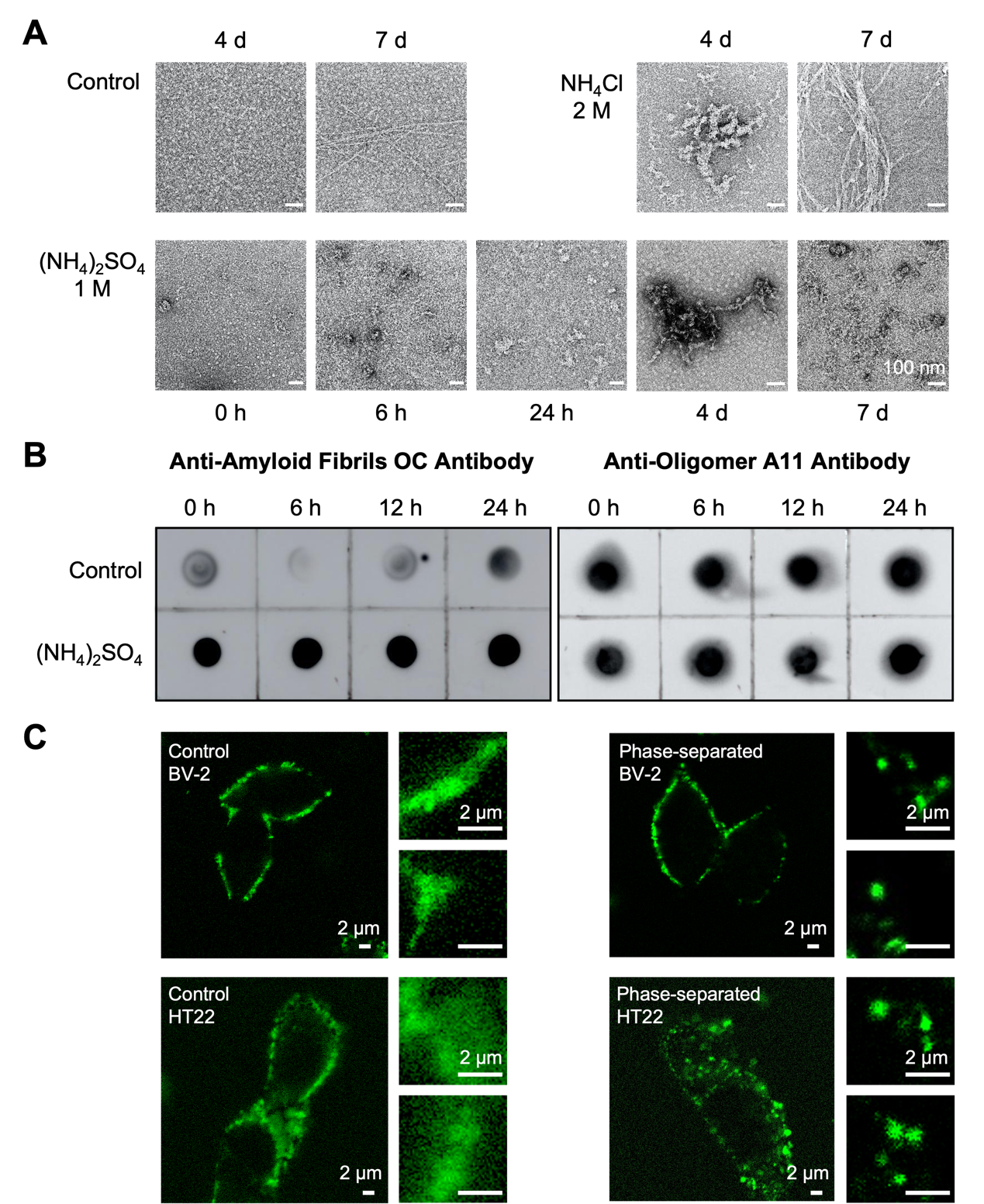


**Figure S5**: A) Amyloid fibrils formation was observed from negative-staining TEM images of 200 µM Aβ_42_Os in 10 mM phosphate buffer, 10 mM NaCl, 0.2% SDS, 0.02% NaN_3_, pH 7.4 containing 2 M ammonium chloride or 1 M ammonium sulfate. Samples were incubated quiescently at 37°C. B) Amyloid fibrillation and oligomerization of Aβ_42_ observed by dot blots immunolabeled with anti-amyloid fibrils OC antibody and anti-oligomer A11 antibody. C) Confocal images of 10 μM Aβ_42_Os in 10 mM phosphate buffer, 10 mM NaCl, pH 7.4 containing 0.2% (w/v) SDS in the absence and presence of 6.4 mM ammonium sulfate in both BV-2 and HT22 cells.
